# Ubiquitin-Specific Protease 29 Regulates Cdc25A-Mediated Tumorigenesis

**DOI:** 10.3390/ijms22115766

**Published:** 2021-05-28

**Authors:** Arun Pandian Chandrasekaran, Sang Hyeon Woo, Neha Sarodaya, Byung Ho Rhie, Apoorvi Tyagi, Soumyadip Das, Bharathi Suresh, Na Re Ko, Seung Jun Oh, Kye-Seong Kim, Suresh Ramakrishna

**Affiliations:** 1 Graduate School of Biomedical Science and Engineering, Hanyang University, Seoul 04763, Korea; aruask.iq@gmail.com (A.P.C.); tkdgus78902@gmail.com (S.H.W.); neyha19@gmail.com (N.S.); aaa0712@hanyang.ac.kr (B.H.R.); apoorvityagi09@gmail.com (A.T.); smdpdas6@gmail.com (S.D.); bharathi.suri@gmail.com (B.S.); 2 Biomedical Research Center, Asan Institute for Life Sciences, Seoul 05505, Korea; nareko.amc@gmail.com; 3 Department of Nuclear Medicine, Asan Medical Center, University of Ulsan College of Medicine, Seoul 05505, Korea; 4 College of Medicine, Hanyang University, Seoul 04763, Korea

**Keywords:** apoptosis, cell cycle, deubiquitinase, oncogenic transformation, proteolysis, tumor model, ubiquitination

## Abstract

Cell division cycle 25A (Cdc25A) is a dual-specificity phosphatase that is overexpressed in several cancer cells and promotes tumorigenesis. In normal cells, Cdc25A expression is regulated tightly, but the changes in expression patterns in cancer cells that lead to tumorigenesis are unknown. In this study, we showed that ubiquitin-specific protease 29 (USP29) stabilized Cdc25A protein expression in cancer cell lines by protecting it from ubiquitin-mediated proteasomal degradation. The presence of USP29 effectively blocked polyubiquitination of Cdc25A and extended its half-life. CRISPR-Cas9-mediated knockdown of *USP29* in HeLa cells resulted in cell cycle arrest at the G0/G1 phase. We also showed that *USP29* knockdown hampered Cdc25A-mediated cell proliferation, migration, and invasion of cancer cells in vitro. Moreover, NSG nude mice transplanted with USP29-depleted cells significantly reduced the size of the tumors, whereas the reconstitution of Cdc25A in USP29-depleted cells significantly increased the tumor size. Altogether, our results implied that USP29 promoted cell cycle progression and oncogenic transformation by regulating protein turnover of Cdc25A.

## 1. Introduction

Recent findings in the fields of cancer and cell cycle regulation have highlighted the coordination of cell proliferation and DNA replication mechanisms [1,2,3,4]. Cell division cycle 25A (Cdc25A) is an important member of the Cdc25 phosphatase family of proteins and controls cell cycle progression by preventing cyclin-dependent kinase (CDK) phosphorylation and triggering the formation of cyclin–CDK complexes [5]. The Cdc25A protein is expressed highly in a variety of human cancer types and is frequently associated with high-grade tumors and confers poor prognosis [6,7]. Likewise, overexpression of Cdc25A in various cancers disrupts the cell cycle [8,9] and results in tumorigenesis and genomic instability, while inhibition of Cdc25A leads to cancer cell apoptosis [10,11]. Thus, Cdc25A is a potential candidate protein contributing to progression of tumors by regulating the cancer cell cycle.

In cancer cells, the rate of Cdc25A protein turnover is controlled by the ubiquitin proteasomal pathway. One study found that βTRCP directs Cdc25A to proteasome-mediated degradation during the S and G2 phases of the cell cycle [12]. Several reports have investigated the ubiquitination of the Cdc25A protein during the cell cycle [12,13,14], but the antagonism of ubiquitination by deubiquitinases also impacts the rate of Cdc25A protein turnover during cell division. Several deubiquitinating enzymes (DUBs) are involved in cell cycle regulation and cancer progression by stabilizing their target substrates [15,16,17]. Generally, DUBs are proteases that protect their target substrates from ubiquitin-mediated proteasomal degradation. To date, four mechanisms have been identified for DUBs: (i) ubiquitin precursor molecule processing, (ii) ubiquitin molecule recycling, (iii) polyubiquitin chain degradation, and (iv) reversal of ubiquitin conjugation. By harnessing these mechanisms, DUBs can rescue the target protein from proteasomal- and lysosomal-dependent degradations. Several studies have reported that DUBs can regulate cell functions such as cell cycle [18] and tumor progression [19]. Therefore, researchers are focusing on therapeutic intervention in DUB activity in the search for new anticancer drugs.

Previously, Dub3 was reported to be the deubiquitinating enzyme for Cdc25A as it acts to regulate the cancer cell cycle and tumor progression [20]. Recently, we performed a genome-wide screening for ubiquitin-specific proteases (USPs) associated with Cdc25A and found that USP3, USP7, and USP29 were the critical deubiquitinases regulating the protein stability of Cdc25A [21,22]. However, the role of USP29 in regulating Cdc25A protein stability and its function during cancer progression have not been studied. In the present study, we demonstrated that USP29 acted as a deubiquitinase to stabilize the Cdc25A protein by rescuing it from ubiquitin-mediated protein degradation. We also showed that USP29 played a critical role in Cdc25A-mediated regulation of the cell cycle in cancer cell lines and in tumor progression.

## 2. Results

### 2.1. USP29 Regulates Cdc25A Protein Stability

**Figure 1:**
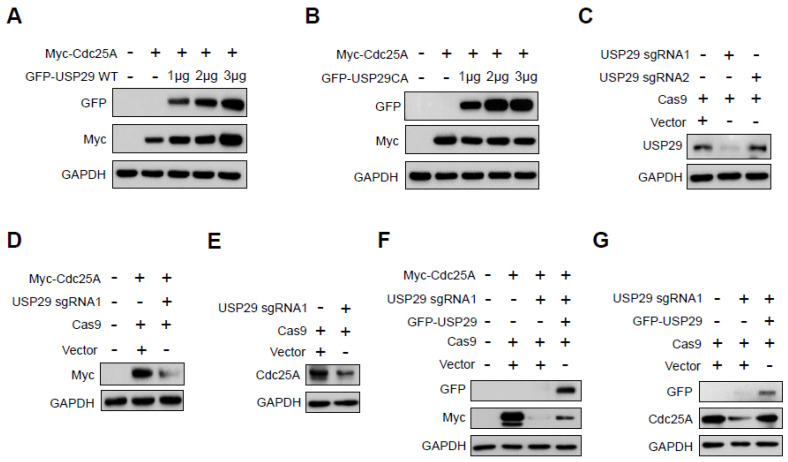
USP29 stabilizes Cdc25A protein expression. (**A**,**B**) HEK293 cells were co-transfected with Myc-Cdc25A and wild-type (WT) GFP-USP29 or GFP-USP29 C294A (CA) in increasing concentrations. Western blots were performed with the indicated antibodies. (**C**) HeLa cells were co-transfected with sgRNAs (sgRNA1 and sgRNA2) targeting *USP29* and Cas9, and *USP29* knockdown was assessed using Western blot analyses. (**D**) HEK293 cells were co-transfected with sgRNA1 targeting *USP29* and Cas9 along with Myc-Cdc25A. The effect of *USP29* knockdown on Myc-Cdc25A was assessed using Western blot analyses. (**E**) HeLa cells were transfected with sgRNA1 targeting *USP29* and Cas9, and the effect of *USP29* knockdown on endogenous Cdc25A protein expression was assessed using Western blot analyses. (**F**) HEK293 cells were transfected with Myc-Cdc25A or co-transfected with sgRNA1 targeting *USP29* and/or GFP-USP29, and exogenous expression of Myc-Cdc25A was assessed using Western blotting with the indicated antibodies. (**G**) HeLa cells were transfected with sgRNA1 targeting *USP29* and/or GFP-USP29 to assess endogenous Cdc25A protein expression. Western blots were performed using the indicated antibodies.

To examine the regulation of Cdc25A protein stability by USP29, we monitored Cdc25A expression in HEK293 cells cultured in the presence of an increasing concentration of USP29. As shown in Figure 1A, overexpression of USP29 was associated with increased expression of the Cdc25A protein. In contrast, ectopic expression of catalytically deficient mutant USP29 (USP29CA) was unable to stabilize the Cdc25A protein (Figure 1B), suggesting that the regulation of Cdc25A stability by USP29 was dependent on the DUB activity of USP29.

We further examined Cdc25A protein expression by transiently knocking down the *USP29* gene using a CRISPR-Cas9 system. To this end, we used two sets of sgRNAs targeting the *USP29* gene, namely, sgRNA1 and sgRNA2 [22], and assessed USP29 knockdown using Western blotting with an anti-USP29 antibody. The expression of the endogenous USP29 protein was downregulated significantly when *USP29* was targeted by sgRNA1 but not when targeted by sgRNA2 (Figure 1C). Furthermore, depletion of *USP29* by sgRNA1 downregulated both exogenous (Figure 1D) and endogenous (Figure 1E) Cdc25A protein expression.

To confirm the stabilizing effect of USP29 on the Cdc25A protein, USP29-depleted HEK293 cells were reconstituted with ectopically transfected USP29, and the expression of Cdc25A was analyzed. Our results showed that overexpression of GFP-USP29 in USP29-depleted cells re-established both exogenous (Figure 1F) and endogenous (Figure 1G) expression of the Cdc25A protein.

### 2.2. USP29 Interacts and Co-Localizes with Cdc25A

**Figure 2:**
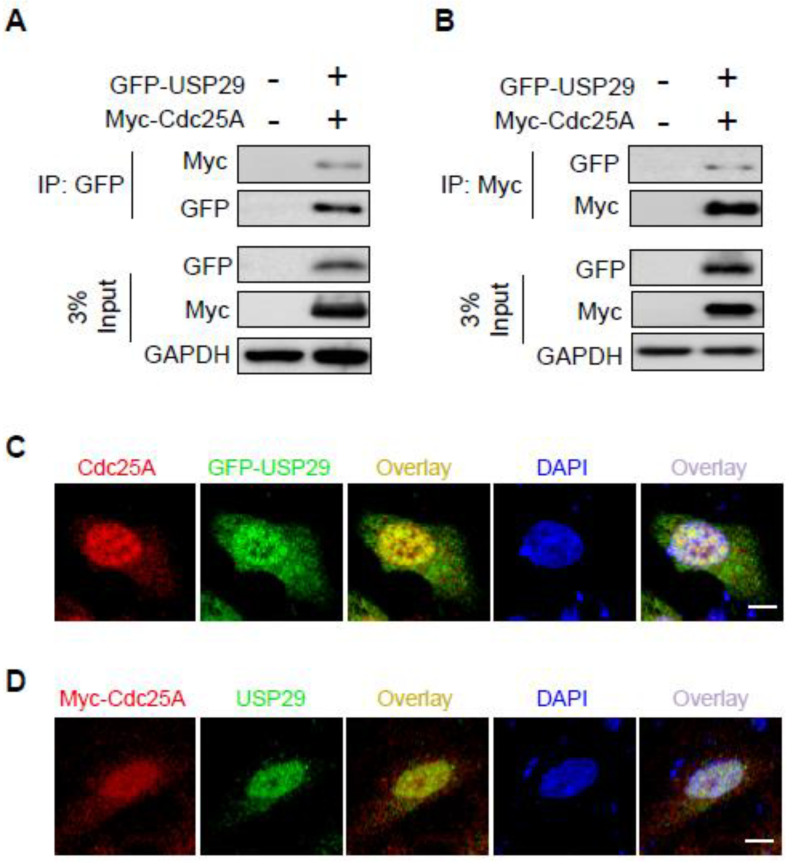
USP29 binds to and co-localizes with Cdc25A. (**A**,**B**) HEK293 cells were transfected with the indicated plasmids to investigate the interactions between GFP-USP29 and Myc-Cdc25A. (**C**,**D**) HeLa cells were transfected with either GFP-USP29 or Myc-Cdc25A, and the cells were immunostained to assess co-localization of USP29 and Cdc25A. Both USP29 and Cdc25A antibodies are produced by the same species, and thus we transfected either GFP-USP29 or Myc-Cdc25A in HeLa cells and immunostained with GFP and Cdc25A or Myc and USP29-specific antibodies. Scale bar = 20 µm.

To further investigate the physical association of Cdc25A and USP29, HEK293 cells were co-transfected with Myc-Cdc25A and GFP-USP29 and subjected to immunoprecipitation with anti-Myc or -GFP antibodies, followed by Western blotting with reciprocal antibodies. Co-immunoprecipitation results showed that Myc-Cdc25A strongly bound to GFP-USP29 and vice versa (Figure 2A,B).

Next, we confirmed the localization of Cdc25A and USP29 HeLa cells. Immunofluorescence staining using specific antibodies revealed that Cdc25A and USP29 were predominantly localized in the nucleus, although a small proportion of the proteins were localized in the cytoplasm (Figure 2C,D). Thus, our data indicated that Cdc25A and USP29 bound to each other and were co-localized.

### 2.3. USP29 Deubiquitinates and Extends the Half-Life of Cdc25A

**Figure 3:**
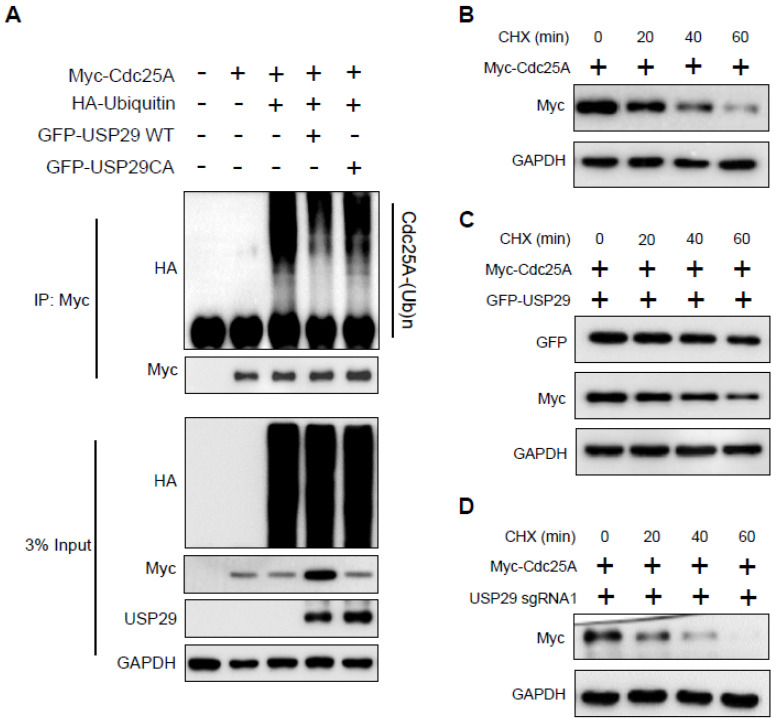
USP29 deubiquitinates and extends the half-life of Cdc25A. (**A**) HEK293 cells were transfected with the indicated plasmids to analyze the deubiquitinating activity of USP29 on Cdc25A. (**B**–**D**) HEK293 cells were transfected with only Myc-Cdc25A (**B**) and in the presence of USP29 (**C**) or sgRNA1 targeting *USP29* (**D**) and treated with CHX (100 µg/mL) for the indicated time periods. Western blot analyses were performed with the indicated antibodies to determine the half-life of Cdc25A.

To investigate the mechanism of Cdc25A stabilization by USP29, we analyzed the ubiquitination status of Cdc25A in the presence of USP29. The HEK293 cells were transfected with Myc-Cdc25A, GFP-USP29, and GFP-USP29CA along with HA-ubiquitin. Immunoprecipitation was performed using an anti-Myc antibody, and the presence of ubiquitinated Cdc25A was detected by immunoblotting with anti-HA. As shown in Figure 3A, wild-type USP29 reduced the ubiquitination of Cdc25A, whereas the catalytic mutant USP29 was unable to do so.

In the cycloheximide chase experiment, we analyzed Cdc25A protein turnover in the presence and absence of USP29. Our results demonstrated that the half-life of Cdc25A is around 20–30 min (Figure 3B). However, the overexpression of USP29 prolonged the half-life of the Cdc25A protein (Figure 3C), whereas USP29-depleted cells caused a severe reduction in half-life of Cdc25A (Figure 3D). Altogether, these findings indicated that USP29 deubiquitinated Cdc25A by preventing it from ubiquitin-mediated proteasomal degradation and extending its half-life.

### 2.4. Loss of USP29 Leads to Cdc25A-Mediated Cell Cycle Arrest and Induces Apoptosis

**Figure 4:**
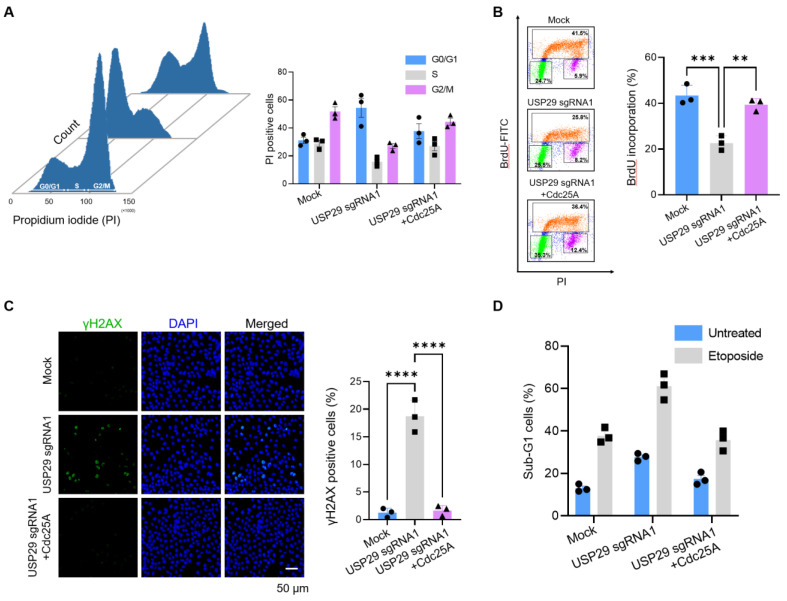
*USP29* knockdown leads to Cdc25A-mediated cell cycle arrest and induces apoptosis. (**A**) HeLa cells were transfected with the indicated plasmids, and the status of the cell cycle was determined by the direct measurement of DNA content using flow cytometry after staining with propidium iodide (PI). (**B**) HeLa cells were transfected with the indicated plasmids and incubated with BrdU to analyze incorporation into the DNA by flow cytometry. (**C**) HeLa cells were transfected with the indicated plasmids and stained with anti-γH2AX antibody and immunofluorescence analyses were performed. Scale bar = 50 µm. (**D**) HeLa cells were transfected with the indicated plasmids and treated with or without etoposide (5 µM) for 12 h to identify the sub-G1 cells by flow cytometry. All data points represent the mean ± standard deviation of three independent experiments (** *p* < 0.01, *** *p* < 0.001, and **** *p* < 0.0001).

Since Cdc25A is known to regulate mitosis and interphase progression, we used HeLa cells to investigate the role of Cdc25A in USP29 depletion. Cell cycle distribution was analyzed by PI staining at 48 h post transfection. As shown in Figure 4A, USP29-depleted cells showed an impaired G1-S phase transition that resulted in an accumulation of cells at the G0/G1 phase compared with the mock control. In contrast, when USP29-depleted cells were reconstituted with ectopically transfected Cdc25A, a lower percentage of cells was arrested in the G0/G1 phase compared to USP29-depleted cells (Figure 4A). Next, BrdU staining was performed to assess the S phase distribution of cells. Consistent with our previous results, the percentage of USP29-depleted cells that entered the S phase was lower than for the mock control cells (Figure 4B). Moreover, when USP29-depleted cells were reconstituted with Cdc25A, the incorporation of BrdU increased, indicating that Cdc25A allows the cells to accumulate in the S phase (Figure 4B).

Next, we visualized the effect of USP29 on DNA damage by performing immunocytochemistry using anti-phospho-H2AX (γH2AX). We observed significantly greater γH2AX expression in cells transfected with sgRNA1 targeting *USP29* compared to the mock control (Figure 4C). In contrast, in USP29-depleted cells reconstituted with ectopically transfected Cdc25A, DNA damage was reduced, and fewer γH2AX immune-positive cells were noted (Figure 4C). In addition, sgRNA1-mediated transient knockdown of *USP29* in HeLa cells increased apoptosis, as indicated by the increased sub-G1 population, whereas the addition of Cdc25A to the USP29-depleted cells reduced apoptosis compared to the parental control (Figure 4D). Thus, our results indicated that USP29 played a crucial role in regulating Cdc25A stability for proper cell cycle progression and prevented apoptosis in cancer cell lines.

### 2.5. Knockdown of USP29 Reduces Cdc25A-Dependent Cancer Growth In Vitro and In Vivo

**Figure 5:**
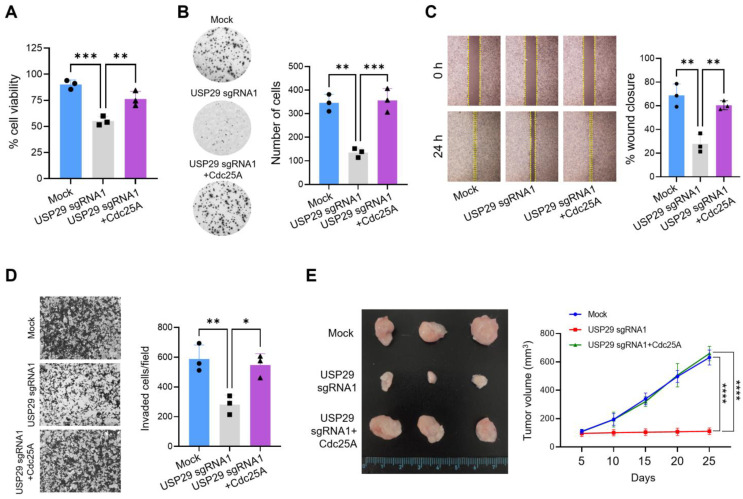
*USP29* knockdown inhibits Cdc25A-mediated cancer cell growth in vitro and in vivo. (**A**) HeLa cells were transfected with the indicated plasmids, and cell viability was assessed. (**B**) HeLa cells were transfected with the indicated plasmids, and a soft agar colony formation assay was performed. (**C**) HeLa cells were transfected with the indicated plasmids, and the wound healing efficiency was analyzed. (**D**) A Matrigel-assisted invasion assay was performed in HeLa cells transfected with the indicated plasmids. (**E**) Mock control, *USP29* knockdown, and *USP29* knockdown HeLa cells in which Cdc25A had been reconstituted were implanted into NSG nude mice for in vivo experiments. Tumor volume was measured and represented graphically. All data points represent the mean ± standard deviation of three independent experiments (* *p* < 0.1, ** *p* < 0.01, *** *p* < 0.001, and **** *p* < 0.0001).

To gain insights into the mechanisms of carcinogenesis-related USP29 activity, we investigated whether USP29 can modulate Cdc25A oncogenic functions in cancer cells. HeLa cells with transient sgRNA1-mediated knockdown of *USP29* showed a significant reduction in the percentage of cell viability compared with the mock control (Figure 5A). Additionally, an anchorage-independent colony formation assay demonstrated that USP29-depleted cells formed fewer colonies compared with the mock control (Figure 5B). However, the rate of cell viability and colony formation recovered when USP29-depleted cells were reconstituted with Cdc25A (Figure 5A,B). Similarly, we studied the effect of USP29 on cell migration and invasion in HeLa cells. Compared with the mock control, USP29-depleted cells exhibited a significant delay in cell migration in an assay of wound healing (Figure 5C) and showed a lower invasion rate (Figure 5D). However, cell migration and invasiveness recovered when USP29-depleted cells were reconstituted with Cdc25A (Figure 5C,D). Collectively, our data indicated that USP29 regulated oncogenic functions of Cdc25A in HeLa cells and can promote tumorigenesis.

To corroborate the USP29-mediated effect on Cdc25A-dependent oncogenic transformation in vivo, we transplanted USP29-depleted HeLa cells, USP29-depleted cells reconstituted with Cdc25A, or mock control cells into NSG mice and measured tumor volume on alternate days for four weeks. In NSG mice transplanted with USP29-depleted cells, tumor volume was significantly reduced compared with those in mice that received mock control cells and expressed USP29 at endogenous levels. On the other hand, transplantation of USP29-depleted cells reconstituted with Cdc25A led to an increased tumor volume (Figure 5E). Taken together, our study results suggested that USP29 facilitated tumor metastasis and progression and exerted its effect by stabilizing and deubiquitinating Cdc25A.

## 3. Discussion

Progression of the eukaryotic cell cycle is tightly maintained by the recurrent expression of regulatory proteins. These regulatory proteins are expressed in a cyclic manner when their functions are needed for cellular processes, including cancer progression. This recurring protein expression is governed largely by the ubiquitin proteasome system; however, in recent years, several studies have revealed additional roles for DUBs in various cellular processes [23,24,25]. A few reports have revealed the involvement of DUBs in all phases of the cell cycle [26]. As we are just beginning to decipher DUB-mediated cell cycle control, identifying novel DUB functions is essential to study the molecular mechanisms of cell cycle regulatory proteins.

In general, DUBs control the progression of the cell cycle and cancer by regulating the activity or stability of target proteins. In this study, we found that USP29 stabilized Cdc25A protein expression and regulated Cdc25A-mediated cell cycle progression and tumorigenesis. Previous studies, including our own recent work, identified DUBs (Dub3, USP3, and HAUSP) involved in Cdc25A-dependent cell cycle and tumor progression [20,21,22]. However, we reported the screening of functional DUBs for Cdc25A using a CRISPR-Cas9 system: a CRISPR-Cas9-based DUB screening has many advantages over conventional siRNA-based screening. Conventional siRNA-based screening methods sometimes cause restoration of the target gene function and result in only partial repression. Identification of deubiquitinating enzymes for Cdc25A using CRISPR-Cas9-based methods is an important finding, as USP29 was previously shown to regulate the expression of the Claspin protein that is involved in DNA replication and cell cycle arrest [27]. As a checkpoint adaptor protein, Claspin directly bridges the ATR/Chk1 pathway, which is the hallmark of tumor progression. We believe that USP29 inhibitors hold promise as therapeutic agents to treat cancer. Given the involvement of USP29 in directly regulating cell cycle progression, a compound that inhibits the expression of USP29 might also sensitize cells to other chemotherapeutic agents.

In conclusion, our data indicated that USP29 acted as a stabilizer of the Cdc25A protein and regulated Cdc25A turnover through its deubiquitinating activity and extended the half-life of Cdc25A in vitro. Finally, we demonstrated that USP29 was critical in promoting Cdc25A-dependent cell cycle progression and tumor formation in vivo. This new insight into the role of USP29 in regulating the cancer cell cycle in tumorigenesis opens new avenues for treatment of cancer.

## 4. Materials and Methods

### 4.1. Cell Culture and Treatments

Human embryonic kidney (HEK293) and cervical cancer (HeLa) cells (ATCC, Manassas, VA, USA) were maintained under standard conditions in DMEM (GIBCO, Waltham, MA, USA) supplemented with 10% fetal bovine serum (GIBCO, Waltham, MA, USA) and 1% penicillin and streptomycin (GIBCO, Waltham, MA, USA) in 5% CO_2_ at 37 °C in a humidified atmosphere. Cells were incubated with the indicated concentrations of cycloheximide (CHX) (Sigma-Aldrich, St. Louis, MO, USA), bromodeoxyuridine (Biolegend, San Diego, CA, USA), or etoposide (Sigma-Aldrich, St. Louis, MO, USA).

### 4.2. Plasmids, Antibodies, and Reagents

Full-length HA-Cdc25A (a kind gift from Dr Zhao-Qi Wang, Friedrich-Schiller University of Jena, Jena, Germany) was subcloned into the pcDNA 3.1 6XMyc-vector. GFP-USP29 and GFP-USP29 C294A were a kind gift from Dr. Han Liu (Dalian Medical University, Liaoning, China). The HA-ubiquitin (#18712) construct was purchased from Addgene (Watertown, MA, USA), and Cas9-2A-mRFP-2A-PAC was purchased from Toolgen (Geumcheon-gu, Seoul, South Korea). The antibodies used in our study were as follows: anti-Cdc25A (55031-1-AP) was purchased from Proteintech (Rosemont, IL, USA), anti-USP29 (HPA021064) from Sigma (St. Louis, MO, USA), anti-BrdU (339811) from Biolegend (San Diego, CA, USA), and anti-Myc (sc-40), anti-GFP (sc-9996), anti-HA (sc-7392) and anti-GAPDH (sc-32233) from Santa Cruz Biotechnology (Dallas, TX, USA).

### 4.3. Cas9 and sgRNA Constructs

The plasmid encoding Cas9-2A-mRFP-2A-PAC (puromycin N-acetyl-transferase and puromycin resistance gene) and sgRNA vectors were purchased from Toolgen (Geumcheon-gu, Seoul, South Korea). The sgRNA target sequences were designed using bioinformatics tools [22] (www.broadinstitute.org, accessed on 21 March 2018) and cloned into the vectors as previously described [28]. The oligonucleotides used to construct sgRNA plasmids are as follows: *USP29* sgRNA1 5′ ACAAAAGGAAATTAAACTGG 3′ and *USP29* sgRNA2 5′ GAGCAACAACATTAGAAGTG 3′.

### 4.4. Transfection

Transient transfection was performed in HeLa and HEK293 cells using Lipofectamine 2000 (Invitrogen, Carlsbad, CA, USA) following the manufacturer’s protocol. For generation of transient *USP29* knockdown, sgRNA1 targeting *USP29* was transfected into HeLa cells along with Cas9-endonuclease. The transfected cells were puromycin selected (1.5 µg/mL) and used for further experiments. Similarly, the mock control was generated by transfecting Cas9 and empty vector into HeLa cells. The transfected cells were puromycin selected as mentioned above and used for experiments.

### 4.5. Immunoprecipitation

For the immunoprecipitation assay, HEK293 cells were transfected with the indicated plasmids for 48 h and lysed with RIPA buffer (50 mM Tris pH 7.6, 150 mM NaCl, 2 mM EDTA, 1% Triton X-100, 0.1% SDS and 1 mM PMSF). Pre-cleared cell lysates were incubated with the respective antibodies overnight at 4 °C and immunoprecipitated with 20 μL of protein A/G Sepharose beads (Santa Cruz Biotechnology, Dallas, TX, USA) the next day at 4 °C for 2 h. Immunoprecipitates were washed with lysis buffer containing 50 mM Tris pH 7.6, 150 mM NaCl, 2 mM EDTA, 1% Triton X-100, 0.1% SDS, 1% sodium deoxycholate, and eluted with 2X SDS sample loading buffer (Cat no. S3401, Sigma-Aldrich, St. Louis, MO, USA) containing 4% SDS, 20% glycerol, 10% 2-mercaptoethanol, 0.004% bromophenol blue, and 0.125 M Tris-HCl, pH 6.8. Co-immunoprecipitated proteins were denatured for 5 min at 95 °C, resolved by SDS-PAGE electrophoresis and analyzed by immunoblotting.

### 4.6. Immunofluorescence

The HeLa cells were transfected with the relevant plasmids and seeded onto glass coverslips 48 h later. The next day, the cells were washed with PBS and fixed in 4% paraformaldehyde for 10 min at room temperature, followed by permeabilization with 0.1% Triton™ X-100 for 5 min and incubation with specific primary antibodies diluted in bovine serum albumin overnight at 4 °C. On the following day, the cells were incubated with conjugated secondary antibodies for 1 h, washed with PBS, and mounted on glass slides. The cells were visualized, and images were captured using a Leica fluorescence microscope (Leica, DM5000 B; Leica CTR 5000; Wetzlar, Hesse, Germany).

### 4.7. Deubiquitination Assay

The DUB activity of USP29 on the Cdc25A protein was examined by transfecting the indicated plasmids into HEK293 cells. Then, 48 h post transfection, the cells were treated with MG132 (5 µM) for 4 h and harvested for immunoprecipitation and Western blotting was performed using the indicated antibodies.

### 4.8. Cell Cycle and Apoptosis Assays

Cell cycle analyses were performed by staining HeLa cells with propidium iodide (PI). First, the cells were fixed in ice-cold 70% ethanol and stained with PI (50 µg/mL; Sigma, St. Louis, MO, USA) and RNase A (200 µg/mL, New England Biolabs, MA, USA) in the dark. The cells were analyzed using a FACS flow cytometer (BD FACSCANTO II, BD Biosciences, Franklin Lakes, NJ, USA). For the bromodeoxyuridine (BrdU) incorporation assay, cells were incubated with 100 µg/mL BrdU for 1 h to identify cells in the S phase. After BrdU incorporation, the cells were collected, fixed in 70% ethanol, resuspended in HCl, and then treated with sodium tetraborate to denature DNA for binding to the BrdU epitopes. These cells were co-stained with anti-BrdU antibody (1:250, Biolegend, San Diego, CA, USA) according to the manufacturer’s protocol. The stained cells were analyzed by flow cytometry within 1 h. To measure the sub-G1 population, HeLa cells were transfected with the indicated plasmids and incubated with either DMSO (untreated) or etoposide for 12 h, and cells with sub-diploid (sub-G1) DNA contents were stained with PI and analyzed by flow cytometry.

### 4.9. Cell Viability Assay

The HeLa cells were transfected with mock, sgRNA1 targeting *USP29*, and USP29-depleted cells, which were reconstituted with Cdc25A plasmid in a 96-well plate and cultured in 200 μL/well of culture medium. After 48 h, the cells were incubated with 10 μL of Cell Counting Kit-8 assay reagent (CCK-8; Dojindo Molecular Technologies, Kumamoto, Kyushu, Japan) for 2 h, and the absorbance was measured using a spectrophotometer (Bio-Rad Laboratories, Hercules, CA, USA) at 450 nm. Data were obtained from three independent experiments.

### 4.10. Soft Agar Assay

For the anchorage-independent colony formation assay, 1 mL of equal ratio of 0.75% agarose gel and 1× DMEM was added to the 6-well culture dish and incubated for 45 min at room temperature. Then, about 1 × 10^4^ cells (HeLa cells transfected with mock, sgRNA1 targeting *USP29*, and USP29-depleted cells were reconstituted with Cdc25A plasmid) were resuspended in 500 µL of DMEM containing 500 µL of 0.75% agarose and planted on the top of the solidified agarose in the 6-well plate. Cells were cultured for 15 days at 37 °C in a humidified atmosphere containing 5% CO_2_. After 2 weeks, the colony numbers were counted by staining with crystal violet, and the colonies were photographed. Data were obtained from three independent experiments.

### 4.11. Wound Healing Assay

To assess cell migration, HeLa cells were transfected with mock, sgRNA1 targeting *USP29*, and USP29-depleted cells reconstituted with Cdc25A plasmid and cultured to 90% confluence. A straight scratch was made in the monolayers with a sterile pipette tip to simulate a wound. The suspended cells were washed off with PBS, and the adherent cells were incubated with serum-free medium. The wounded area was captured at 0 and 24 h using a fluorescence microscope (IX71, Olympus, Hachioji, Tokyo, Japan) and ImageJ software (version 1.51j8, University of Wisconsin, Madison, WI, USA) was used to measure the percentage of wound healing. Data were obtained from three independent experiments.

### 4.12. Matrigel Invasion Assay

To determine the invasiveness of the cells, 0.8 µm Transwell chambers (Falcon, Corning, NY, USA) coated with Matrigel (Corning, NY, USA) were seeded with 2.5 × 10^4^ cells suspended in 500 µL of serum-free DMEM and placed in a 24-well plate. Next, 750 µL of complete medium was added to the 24-well chambers and incubated at 37 °C. After 24 h, the invading cells that had migrated toward the complete medium were fixed with ice-cold methanol and stained with crystal violet. The average number of invading cells per well was counted using light microscopy. Data were obtained from three independent experiments.

### 4.13. Xenograft Tumor Experiment

Male NOD SCID γ (NSG) mice (6 weeks old) were used to validate our results in vivo. The animal study was approved by the IACUC. All mice were housed under standard conditions with a 12 h light/dark cycle and free access to food and water. For xenograft experiments, the animals were randomized into three groups (n = 3 per group), and 5 × 10^6^ cells suspended in PBS:Matrigel (1:1, BD Biosciences, Franklin Lakes, NJ, USA) were injected into the subcutaneous tissue on the right flank of the mice. Two co-authors individually estimated the volume of the xenografts every other day for 30 days using the formula *V* = *D* × *d*^2^/2, where *D* is the long axis and *d* is the short axis of the tumor. Upon completion of the experiments, the tumor grafts were harvested and weighed.

### 4.14. Statistical Analyses

All statistical analyses were conducted using GraphPad Prism software (Version 9.0, San Diego, CA, USA). All experiments were performed independently at least three times, and the results are presented as mean ± SD. One-way or two-way ANOVA followed by the Tukey post hoc test was performed to compare three or more groups. A *p* value < 0.05 was considered statistically significant.

## Data Availability

Not applicable.

## Abbreviations

Cdc25A, cell division cycle 25A; DUB, deubiquitinating enzyme; USP, ubiquitin-specific protease; CDK, cyclin-dependent kinase; sgRNA, single-guide RNA; HA, haemagglutinin; HEK293, human embryonic kidney 293; CHX, cycloheximide; and BrdU, bromodeoxyuridine.
